# Antioxidant, Hepatoprotective, and Antidepression Effects of* Rumex tingitanus* Extracts and Identification of a Novel Bioactive Compound

**DOI:** 10.1155/2018/7295848

**Published:** 2018-03-18

**Authors:** Dhekra Mhalla, Karama Zouari Bouassida, Rachid Chawech, Amira Bouaziz, Samar Makni, Lobna Jlaiel, Slim Tounsi, Raoudha Mezghani Jarraya, Mohamed Trigui

**Affiliations:** ^1^Biopesticides Laboratory, Center of Biotechnology of Sfax, University of Sfax, P.O. Box 1177, 3018 Sfax, Tunisia; ^2^Laboratory of Chemistry of Natural Products, Faculty of Sciences of Sfax, P.O. Box 1171, 3000 Sfax, Tunisia; ^3^Analysis Service of the Center of Biotechnology of Sfax, University of Sfax, P.O. Box 1177, 3018 Sfax, Tunisia; ^4^Research Unit “Coastal and Urban Environments”, Sfax Preparatory Engineering Institute, University of Sfax, BP 1172, 3018 Sfax, Tunisia

## Abstract

Over the last few decades,* Rumex* species have been recognized as a promising source of new compounds with numerous pharmacological activities. Therefore, the antioxidant activity of* Rumex tingitanus (R. tingitanus)* leaves extracts was evaluated* in vitro* and then confirmed* in vivo *as well as the antidepressant-like and toxicological effects of the extracts. The ethyl acetate fraction (Rt EtOAcF) followed by hydroalcoholic extract (Rt EtOH-H_2_O) showed a remarkable* in vitro* antioxidant activity. The hydroalcoholic extract (Rt EtOH-H_2_O) showed significant hepatoprotective activity against carbon tetrachloride- (CCl_4_-) induced liver toxicity which is seen from inhibition of the malondialdehyde (MDA) accumulation and enhancement of the liver antioxidant enzymes activities. The Rt EtOH-H_2_O and Rt EtOAcF extracts were able to reduce the immobility time in mice and then elicited a significant antidepressant-like effect. The ethyl acetate fraction (Rt EtOAcF) was purified and resulted in the identification of a new antioxidant component called 4′-*p*-acetylcoumaroyl luteolin. The Rt EtOAcF and the 4′-*p*-acetylcoumaroyl luteolin revealed a strong antioxidant activity using DPPH test with IC_50_ of 11.7 ± 0.2 and 20.74 ± 0.6 *μ*g/ml, respectively, and AAI of 3.39 and 1.92 better than that of BHT, used as control.

## 1. Introduction

The extraction and characterization of antioxidant compounds derived from natural sources could be a solution to treat a variety of injuries and diseases caused by oxidative stress such as depression and hepatotoxicity. Depressive disorders, characterized by a loss of energy, anhedonia, sleep disturbances, and the decreases in the ability to think and concentrate are considered as actually as an example of the most common mental disorders worldwide [1, 2]. It was reported that oxidative stress is one of the biological processes involved in the development of the depressive symptoms [3]. The excessive accumulation of the reactive oxygen species (ROS) and the deficiency in the antioxidant enzymes induce a major cellular damage leading to diseases development such as the depressive disorder [2, 4].

The reactive oxygen species (ROS), active forms of oxygen, cause damage of cellular macromolecules (protein, lipids, and DNA). To prevent the undesirable effect of the ROS, human organisms have developed an antioxidant defense system. Catalase (CAT), superoxide dismutase (SOD), and glutathione peroxidase (GPx) are examples of the most implicated enzymes in the ROS conversion to stable molecules, such as water and O_2_ [4]. One of the vital organs which regulates this metabolic process and maintains homeostasis is the liver. Any injury induced by ROS or xenobiotics to hepatocytes causes hepatotoxicity and liver disorders [5]. In the literature, various chemical and xenobiotic agents are known for their ability to induce hepatotoxicity. The carbon tetrachloride (CCl_4_) is one of the hepatotoxins causing hepatotoxicity similar to human cirrhosis [6]. Over the last decades, herbs have emerged as prominent alternatives to fight oxidative stress, considering that plants are important sources of flavonoids, tannins, polyphenols, anthraquinones, stilbenoids, and steroids [2].

Given the aforementioned beneficial effects of plants metabolites, the genus* Rumex* has been identified as a promising pharmacological candidate. Further research aiming to investigate the hepatoprotective effects, toxicity, and other pharmacological activities should be developed according to Vasas et al. [7]. Approximately 200 species of* Rumex* are spread worldwide, among which only around 50 species have been studied so far and little phytochemical information and pharmacological information are available. The phytochemical screening of* Rumex* species revealed their richness in anthraquinones and flavonoids but till today only few compounds are isolated [8]. The genus includes edible plant species used in traditional medicine for inflammation, blood purification, and constipation or for their purgative and tonic effects. The wild edible plant* Rumex tingitanus* L.* (R. tingitanus)* which grows in spring is consumed fresh or cooked [9]. Therefore, this species was subject of some experimental work in order to (i) determine the antioxidant activities of* R. tingitanus *leaves extracts* in vitro*, (ii) evaluate the hepatoprotective activity of Rt EtOH-H_2_O extract against CCl_4_-induced hepatic oxidative damage in rats, (iii) investigate toxicological and antidepressant-like properties of* R. tingitanus* leaves extracts, and (iv) isolate and elucidate the structure of the antioxidant component(s) from the most active extract.

## 2. Materials and Methods

### 2.1. Extract Preparation, Separation, and Isolation of Secondary Metabolites

Leaves powder of* R. tingitanus *(1.5 Kg) was extracted during 48 h by maceration with 6 L of ethanol (80%). Then, the solvent was evaporated at 40°C and the remaining aqueous phase was lyophilized to produce 304.5 g of hydroalcoholic extract (Rt EtOH-H_2_O extract). The resulting hydroalcoholic extract was sequentially fractionated by liquid/liquid chromatography with* n*-hexane followed by ethyl acetate to obtain an* n*-hexane fraction (Rt HexF), ethyl acetate fraction (Rt EtOAcF), and water fraction (Rt WF), respectively. The organic phases were filtered and evaporated at 40°C and the aqueous phase was filtered and lyophilized. These extracts and fractions were stored at 4°C.

The ethyl acetate fraction was fractionated by silica gel column chromatography (60 Å, 70–200 *μ*m), using an increasing-polarity solvent [10]. The isolated subfractions were evaluated for their antioxidant activity. Fraction showing the highest antioxidant activity was submitted to further purification using a dichloromethane-methanol binary solvent gradient. The purity of the isolated compound was over 98.7% checked by LC-MS/MS analysis (Figure 4). An Agilent MSD Ion Trap XCT mass spectrometer equipped with an ESI ion source was used. The mass spectrometer was operated in a negative mode with source voltage of 3.5 kV. This compound was identified through the analysis of its spectroscopic data of ^1^H- and ^13^C-NMR, IR, UV, and MS.

*The 4*′*-p-acetylcoumaroyl Luteolin*. It is a white amorphous powder,* UV* (MeOH) *λ*max (nm): 254, 268, 360;* IR (cm*^−1^): 3500, 3255, 2980, 2820, 1719, 1625, 1650, 1510, 1390, 1255. ESI-MS:* m/z* 473 [M−H]^−^ (C_26_H_17_O_9_).

^1^ *H-NMR (CD*_3_ *OD, 400 MHz)*. *δ* (ppm) 6.52 (1H, s, H-3), 6.23 (1H, d (2.3 Hz), H-6), 6.92 (1H, d (2.3 Hz), H-8), 7.04 (1H, d (1.8 Hz), H-2′), 7.36 (1H, d (7.8 Hz), H-5′), 7.45 (1H, dd (7.8, 1.8 Hz), H-6′), 6.22 (1H, d (15.3 Hz), H-1′′), 7.53 (1H, d (15.3 Hz), H-2′′), 7.44 (2H, d (7.2 Hz), H-4′′, H-8′′′′), 6.79 (2H, d (7.2 Hz), H-5′′, H-7′′), 1.27 (3H, s, H_Ac_).

^13^ *C-NMR (CD*_3_ *OD, 100 MHz)*. *δ* (ppm) 164.99 (C, C-1), 102.15 (C, C-3), 182.44 (C, C-4), 161.70 (C, C-5), 98.82 (CH, C-6), 164.49 (C, C-7), 93.18 (CH, C-8), 157.99 (C, C-9), 104.09 (C, C-10), 122.32 (C, C-1′), 112.89 (CH, C-2′), 145.70 (C, C-3′), 149.50 (C, C-4′), 115.45 (CH, C-5′), 119.02 (CH, C-6′), 169.96 (C, C=O), 114.38 (CH, C-1′′), 145.68 (CH, C-2′′), 126.89 (C, C-3′′), 121.60 (2CH, C-4′′, C-8′′), 115.18 (2CH, C-5′′, C-7′′), 150.05 (C, C-6′′), 29.45 (CH_3_, Acetyl), 178.41 (C, Acetyl).

### 2.2. Preliminary Phytochemical Screening

Qualitative chemical tests for various phytoconstituents were carried out to highlight the main families of secondary metabolites present in all the extracts of* R. tingitanus *as reported by [11].

### 2.3. Secondary Metabolites Determination

The total polyphenols content (TPC) was determined by colorimetric method using Folin-Ciocalteu phenol reagent [12] and the results were expressed in mg of gallic acid equivalent/g of sample (mg GAE/g). The content of flavonoids (TF) was estimated by spectrophotometric assay and was expressed in mg of quercetin equivalent/g of sample (mg QE/g) [13].

### 2.4. Pharmacological Activities

#### 2.4.1. Acute Toxicity Study

Mice that weighed between 25 and 39 g were divided into ten groups (*n* = 6 in each). A first control group received distilled water, while the second control group received a solution of Tween-80 (5%). Hydroalcoholic extract (Rt EtOH-H_2_O) was dissolved in distilled water, whereas the ethyl acetate fraction (Rt EtOAcF) was dissolved in Tween-80 (5%). The remaining groups were treated by intraperitoneal injection using different extracts concentrations (100, 150, 300, and 500 mg/Kg BW). Mice were maintained on standard animal diet and water. The animal mortality was tracked for 7 days after treatment [14].

#### 2.4.2. Antidepressant-Like Effect

The antidepression effect was determined by a forced swimming test (FST) used extensively for the antidepressant drugs evaluation. The mice were subjected to noise for one hour and were individually placed into Plexiglass cylinder (diameter: 16 cm; height: 21 cm) containing 11.5 cm of water at 23 ± 2°C. The immobility time was recorded during a short period (6 min test). A decrease in total time immobility indicates an antidepressant-like effect. For that, four groups of six animals (*n* = 6) received 50 and 100 mg/kg BW of the Rt EtOH-H_2_O and Rt EtOAcF, 30 min before the test. The control group received a solution of Tween-80 (5%). Two independent groups treated with clomipramine (CLI) with 25 and 50 mg/kg BW doses served as positive control [15].

#### 2.4.3. Antioxidant Activity

*(1) DPPH Antiradical Activity*. Antiradical activities of Rt EtOH-H_2_O extract and Rt EtOAcF, Rt HexF, and Rt WF fractions were measured by DPPH as reported by Trigui et al. [16]. Briefly, 50 *μ*l of various extract concentrations was added to 2 ml of a DPPH solution (0.04 g/l in methanol) followed by 30 min incubation in the dark at room temperature. Ascorbic acid was used as a positive control. The absorbance was measured at 517 nm against the corresponding blank in triplicate for each extract. The results were expressed as the antioxidant activity index (AAI) using the two following formulae:

(1) I%=Acontrol−AsampleAcontrol×100AAI=Final  concentration  of  DPPHμg/mLIC50μg/ml,

where *A*_control_ is the absorbance of the negative control and *A*_sample_ is the sample absorbance.

IC_50_ was defined as the antioxidant concentration reducing 50% of DPPH free radicals and was then determined for each extract, luteolin and 4′-*p*-acetylcoumaroyl luteolin. According to the AAI values, we considered a poor antioxidant activity when AAI < 0.5, moderate antioxidant activity when 0.5 ≤ AAI ≤ 1, strong antioxidant activity when 1.0 ≤ AAI ≤ 2.0, and very strong antioxidant activity when AAI > 2.0, respectively.

*(2) β-Carotene-Linoleic Acid Bleaching Assay*. The inhibitions of the linoleic acid autooxidation by Rt EtOH-H_2_O extract, Rt EtOAcF, Rt HexF, and Rt WF fractions were evaluated by the method of Trigui et al. [16]. The mixture containing 0.2 mg of *β*-carotene, 20 *μ*l of linoleic acid, and 200 mg of Tween-40 was dissolved in 1 ml of chloroform. After the solvent evaporation, 50 ml of oxygen-bubbled water was added. 5 ml of the obtained emulsion was mixed with 500 *μ*l of each extract and incubated for 2 h at 50°C before measuring absorbance at 470 nm. The butylated hydroxytoluene (BHT) was used as positive control, and a blank was used as a negative control.

The antioxidant activity (Inhibition%) was determined using the following equation:

(2) $$\begin{array}{c} Inhibition\%=\left(\;\frac{A_{\beta \text{-}carotene\;\;after\;\;2\;h\;\;assay}}{A_{initial\;\;\beta \text{-}carotene}}\;\right)\times 100, \end{array}$$

where *A*_*β*-carotene  after  2 h  assay_ and *A*_initial  *β*-carotene_ are the absorbance of *β*-carotene after 2 h assay and the absorbance of *β*-carotene at the beginning of the experiments, respectively. The necessary antioxidant concentration to reduce 50% of the absorbance (IC_50_) was determined. The experimental tests were performed in triplicate for each extract.

*(3) Reducing Power*. The reducing power effect of the Rt EtOH-H_2_O, Rt EtOAcF, Rt HexF, and Rt WF extracts was determined according to Oyaizu [17]. 1 ml of the different extract concentrations (0.0155, 0.0315, 0.125, 0.5, and 1 mg/ml) was mixed with 2.5 ml of phosphate buffer (0.2 M, pH 6.6) and 2.5 ml of potassium ferricyanide [K_3_Fe(CN)_6_] (1%) and incubated at 50°C. After 20 min of incubation, 2.5 ml of a TCA solution (10%) was added to the mixture followed by a centrifugation at 3000 rpm for 10 min. Distilled water (2.5 ml) and 0.5 ml of aqueous solution of FeCl_3_ (1%) were added to the supernatant and then the absorbance was measured at 700 nm against blank. The BHT was used as a positive control. IC_0.5_, which is the concentration of extract that provides 0.5 absorbance, was determined for each extract in triplicate.

#### 2.4.4. *In Vivo* Antioxidant Properties

*(1) Animals*. Albino Wistar rats (males, 130–160 g) were obtained from SIPHAT, Tunisia, and maintained at 22 ± 3°C with 12 h light/12 h dark cycle and relative humidity of 40%. To minimize the effects of stress caused by travel, the rats were acclimated for a week in the laboratory conditions before the experiment. Water and feed were available at will.

*(2) Experimental Design*. The protective effect of the hydroalcoholic leaves extract of* R. tingitanus* (Rt EtOH-H_2_O) against liver damage induced by CCl_4_ was evaluated. The rats were randomly divided into groups of six (*n* = 6):

- Group I (normal control) was treated with 1 ml kg^−1^ of olive oil injected intraperitoneally on day 8.
- Group II (CCl_4_ control) was treated with 1 ml/kg BW of CCl_4_ in olive oil injected intraperitoneally on day 8.
- Group III (extract control) was treated with 250 mg/kg BW of Rt EtOH-H_2_O injected daily intraperitoneally during 8 days.
- Group IV (extract treated) was treated daily with 250 mg/kg BW of the hydroalcoholic extract (Rt EtOH-H_2_O) injected intraperitoneally during 8 days followed by a single dose of CCl_4_ in olive oil at a dose of 1 ml/kg five hours after the last injection.
- Group V (reference control) was treated with 50 mg/kg BW of gallic acid (GA) daily for 8 days by intraperitoneal injection.
- Group VI (reference treated) was treated with 50 mg/kg BW of gallic acid (GA) daily for 8 days by intraperitoneal injection and then a dose of 1 ml/kg of CCl_4_ in olive oil five hours after the last injection.

On day 9, the animals were sacrificed by cervical decapitation and the liver was excised and crushed in 50 mM Tris, 150 mM NaCl buffer (pH 7.4) using an Ultra-Turrax homogenizer. The homogenate was centrifuged at 3000 ×g at 4°C for 15 min and the collected supernatant was frozen at −20°C.

*(3) Estimation of Malondialdehyde (MDA) in Liver Tissue*. Malondialdehyde (MDA) in the liver homogenate was performed as follows: 375 *μ*l of the supernatants, 150 *μ*l of TBS, and 375 *μ*l of TCA-BHT (20% - 1%) were mixed and centrifuged at 1000 ×g for 10 min. 80 *μ*l of HCl (0.6 N) and 320 *μ*l of Tris-TBA [Tris (26 mM) and TBA (120 mM)] solution were mixed with 400 *μ*l of the obtained supernatant and incubated at 80°C for 10 min. The developed color absorbance using 1,1,3,3-tetramethoxypropane as an external standard was measured at 530 nm. Tests were performed in triplicate for each extract and the results were expressed in nmole MDA equivalent formed/mg protein. The protection percentage was calculated using the following formula:

(3) protection%=CCl4  control−extract  treatedCCl4  control−normal  control×100.

*(4) Enzymatic Antioxidant Analyses*. The superoxide dismutase (SOD) activity was measured using the nitroblue tetrazolium (NBT) inhibition according to Beyer Jr. and Fridovich [18]. The catalase (CAT) content was determined colorimetrically from the rate of H_2_O_2_ decomposition used as substrate [19]. The glutathione peroxidase (GPx) activity was analysed colorimetrically using H_2_O_2_ as substrate and the reduced glutathione (GSH) [20].

*(5) Histopathological Studies*. The excised liver tissues were fixed in 10% formaldehyde solution during 24 h. After dehydrating in graded (50–100%) alcohol, the liver tissues were processed by being embedded in paraffin. Liver sections (4-5 *μ*m thickness) were stained with hematoxylin and eosin and examined for histopathological changes. Photomicrographs of each of the liver slices were taken at 200x magnification [21].

### 2.5. Statistical Analysis

All the experiments performed in this study were repeated at least three times and data were reported as means ± standard error of three measurements. All the statistical comparisons were made by means of one-way ANOVA test. Student's* t*-test was performed to determine significant differences between the treatments and the control using SPSS 19 statistical package (SPSS Ltd., Woking, UK). *P* values less than 0.05 were considered as significant.

## 3. Results

### 3.1. Phytochemical Screening

The qualitative distribution of polyphenols, tannins, and flavonoids showed a difference from one extract to another. Moreover, the Rt EtOAcF showed the presence of low quantities of anthraquinones. However, proteins and amino acids were detected only in Rt WF. The hexane fraction contained steroids and cardiac glucosides.

### 3.2. Total Phenolic and Flavonoid Contents

**Figure 1:**
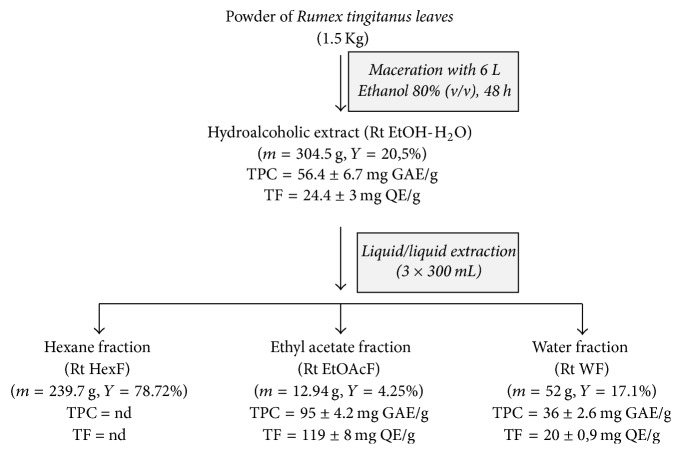
Successive extractions of* Rumex tingitanus* leaves as well as yield (*Y* in %) and content of total polyphenols (TPC) and total flavonoids (TF) in the different extracts.

The quantitative analysis of phenolic and flavonoid contents of* R. tingitanus *extracts is summarized in Figure 1. The amount of these metabolites varied from one extract to another depending on the solvents polarities used for the extraction. The Rt EtOAcF had the highest phenolics concentration (95 ± 4.2 mg GAE/g) compared to Rt EtOH-H_2_O extract (56.4 ± 6.7 mg GAE/g) and Rt WF (36 ± 2.61 mg GAE/g). The Rt EtOAcF also contained the highest flavonoid amounts (119 ± 8 mg QE/g), 4 times more than Rt EtOH-H_2_O extract and Rt WF with 24.4 ± 3 and 20 ± 0.9 mg QE/g, respectively (Figure 1).

### 3.3. Acute Toxicity Study

After a one-week treatment, the hydroalcoholic extract and ethyl acetate fraction of* R. tingitanus* did not show any signs of toxicity up to the dose of 500 mg/kg BW. The mice did not exhibit any common side effects such as mortality, loss of weight, diarrhea, and abnormal behavior during the observation period compared to the control group. Therefore, the Rt EtOH-H_2_O at 250, 100, and 50 mg/Kg BW was used in the* in vivo* investigation for the antioxidant activity and the antidepressant-like effect, respectively.

### 3.4. Antidepressant-Like Effect of* R. tingitanus* Extracts

**Figure 2:**
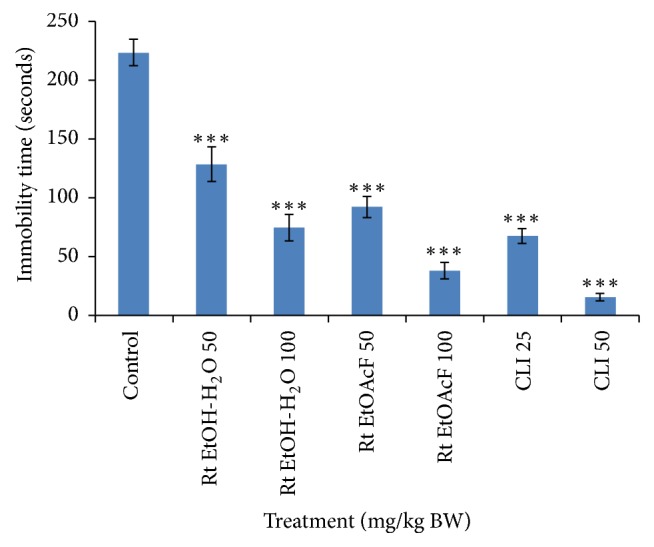
The effect of Rt EtOH-H_2_O and Rt EtOAcF extracts (50 and 100 mg/kg) or clomipramine (CLI) (25, 50 mg/kg) on the immobility time measured in the forced swim test in different groups of mice. The values shown in this figure are mean ± SE. *n* = 6. The statistical test used is Student's* t*-test (*n* = 6). ^*∗*^*P* < 0.05, ^*∗∗*^*P* < 0.01, and ^*∗∗∗*^*P* < 0.001, respectively, compared to the control group.

As shown in Figure 2, the administration of the tested extracts showed significant (*P* < 0.001) decrease in the immobility time compared to the control mice. In fact, Rt EtOAcF and Rt EtOH- H_2_O exhibited an immobility reduction of 83% and 67% using 100 mg/Kg, respectively. These tests clearly prove that the* R. tingitanus* extracts' antidepressant-like effect was similar to the clomipramine treated mice, used as positive control.

### 3.5. Antioxidant Activity

#### 3.5.1. DPPH Antiradical Activity

The antiradical assay was used to study the ability of an extract or a compound to trap free radicals by a hydrogen-donating action [22]. The antiradical activities of* R. tingitanus* extracts, measured by DPPH assay, were found to be concentration-dependent. The Rt EtOAcF and Rt EtOH-H_2_O showed a percentage inhibition of 97% towards DPPH free radical in the presence of 24.4 and 146.3 *μ*g/ml, respectively. Besides, Rt WF showed a 90% inhibition using 146.3 *μ*g/ml and the Rt HexF was less effective. The IC_50_ and AAI values of the different extracts are depicted in Table 1. The most potent antiradical activity was obtained with Rt EtOAcF (IC_50_ = 11.7 ± 0.2 *μ*g/ml) followed by Rt EtOH-H_2_O (IC_50_ = 78.1 ± 1.5 *μ*g/ml) and then by Rt WF (IC_50_ = 81.4 ± 4 *μ*g/ml) and finally by Rt HexF (IC_50_ = 193.8 ± 7.9 *μ*g/ml). The AAI varied from 0.49 (water fraction) to 3.39 for ethyl acetate fraction. A very strong antioxidant activity was observed for the ascorbic acid followed by the ethyl acetate fraction, which were more potent than the BHT. The antiradical activity due to a hydrogen transferring reaction showed a positive correlation with phenolics amount (*R*^2^ = 0,956) and therefore their implication in* R. tingitanus* antiradical capacity.

#### 3.5.2. *β*-Carotene-Linoleic Acid Bleaching Assay

Unsaturated fatty acids, involved in the formation of cell membrane, are susceptible to lipid peroxidation. Several peroxyl radical and by-products were produced, leading to tissue damage and diseases processes such as neurodegenerative diseases, carcinogenesis, and inflammation [23]. So, the* R. tingitanus* extracts' capacity to inhibit the lipid peroxidation of linoleic acid bleaching assay was investigated. The antioxidant activities of Rt EtOAcF and Rt EtOH-H_2_O are dose-dependent and showed an inhibition of 100% and 93.2%, respectively, in the presence of 1.2 mg/ml. Fractions Rt HexF and Rt WF showed an inhibitory effect on lipid peroxidation within the tested concentration range. The antioxidant potential of* R. tingitanus* extracts can be investigated by measuring the IC_50_ values. As shown in Table 1, the Rt EtOAcF (IC_50_ = 320 ± 16 *μ*g/ml) followed by Rt EtOH-H_2_O (IC_50_ = 451 ± 9 *μ*mg/ml) exhibited a promising antioxidant activity.

#### 3.5.3. Reducing Power Assay

The Rt EtOAcF showed a potent reducing capacity with an absorbance of 12.6 at a concentration of 1 mg/ml followed by Rt EtOH-H_2_O and then Rt WF and finally Rt HexF with absorbances of 6.7, 5.1, and 2.8, respectively, at the same concentration. This activity increased in a concentration-dependent way. The determined IC_0.5_ values of the different extracts revealed a high capacity for the Rt EtOAcF similar to that of the BHT used as antioxidant control (Table 1). The reducing power of all extracts from* R. tingitanus* leaves can be presented in the following order: Rt EtOAcF > Rt EtOH-H_2_O > Rt WF > Rt HexF.

#### 3.5.4. *In Vivo *Effects on the Malondialdehyde (MDA) Levels

The protective effect of Rt EtOH-H_2_O extract against liver peroxidative damage induced by CCl_4_ is shown in Table 2. For the CCl_4_-treated rats, the MDA levels increased significantly (*P* < 0.01) compared to the normal control group. Nevertheless, the group treated with 250 mg/kg BW of Rt EtOH-H_2_O significantly reversed (*P* < 0.01) the elevation of the MDA levels by 100% compared to the CCl_4_-treated group. Gallic acid, used as reference, reinstated the MDA formation by 95.2%. Animals treated with Rt EtOH-H_2_O and gallic acid showed similar MDA concentrations compared to normal control group.

#### 3.5.5. Effects on Enzymatic Antioxidant Activities

The interacting network of antioxidant enzymes, especially CAT, SOD, and GPx, has essential roles in the detoxification pathway against ROS. The hepatoprotective effect of Rt EtOH-H_2_O extract against CCl_4_ was presented in Table 2. The enzymes activities (SOD, CAT, and GPx) decreased significantly in the liver tissue of the rats treated with CCl_4_ when compared to the normal control group. However, no significant differences in enzymatic antioxidants levels were observed in animals treated with Rt EtOH-H_2_O compared to the normal control rat values. In the Rt EtOH-H_2_O treated group (Group IV), using 250 mg/kg BW caused the tested parameters to regain their control values. The* R. tingitanus* leaves hydroalcoholic extract showed a protection of 100%, 98.8%, and 96.2% for SOD, CAT, and GPx, respectively, compared to 84.3%, 100%, and 98.1% using gallic acid as reference towards hepatotoxicity induced by CCl_4_.

#### 3.5.6. Histopathological Examinations of the Liver Tissues

**Figure 3:**
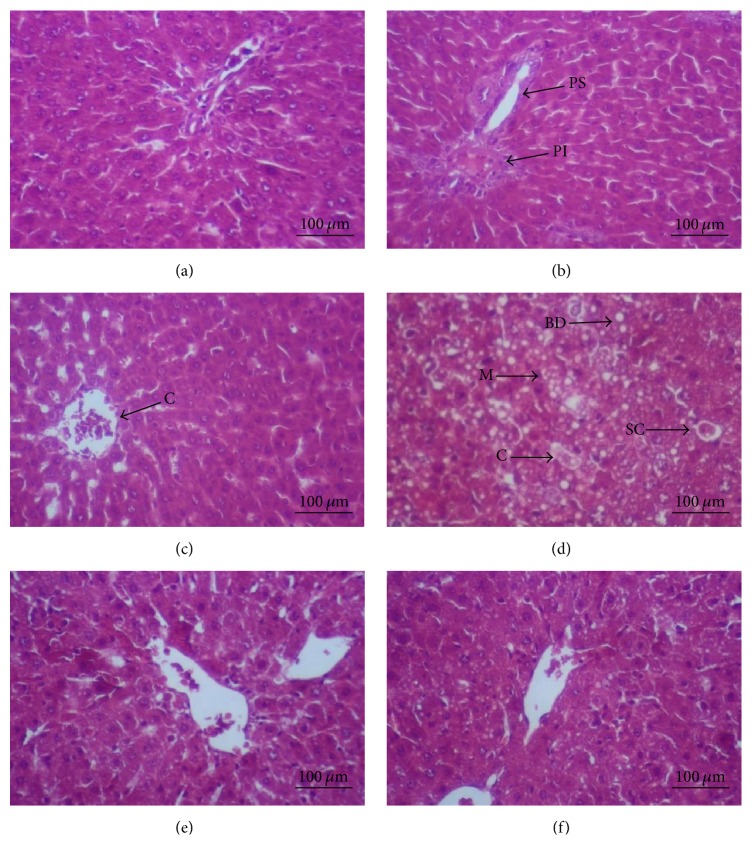
Histological observation on liver tissues of controls and experimental rats. (a) Control group (olive oil); (b) groups that received gallic acid; (c) groups that received Rt EtOH-H_2_O; (d) group treated with CCl_4_; (e) groups treated with the combination GA/CCl_4_, and (f) groups treated with the combination Rt EtOH-H_2_O/CCl_4_. Hematoxylin-eosin method was used to stain the liver sections (magnification, 200x; scale bars, 100 *μ*m). Arrow: CV: central vein; PS: portal spaces; PIN: infiltration in periportal area; (SCN): single cell necrosis; BD: ballooning degeneration; MC: micro and macrovesicular changes; C: congestion.

The liver histopathology of CCl_4_-intoxicated rats, Rt EtOH-H_2_O treated group, and gallic acid reference treated group is illustrated in Figure 3. The histological observation of the control slices showed normal cellular architecture of the liver with distinct hepatocytes, central vein, and portal and sinusoidal spaces. However, the CCl_4_ treatment caused histopathological changes and severe liver injuries including necrosis, ballooning degeneration, micro and macrovesicular changes, and congestion. In the tissue of the treated groups with Rt EtOH-H_2_O and gallic acid, alterations were minimized and the liver injuries were improved. The liver architectural pattern of the rats treated with only Rt EtOH-H_2_O or gallic acid was preserved.

### 3.6. Fractionation and Antioxidant Activity

**Figure 4:**
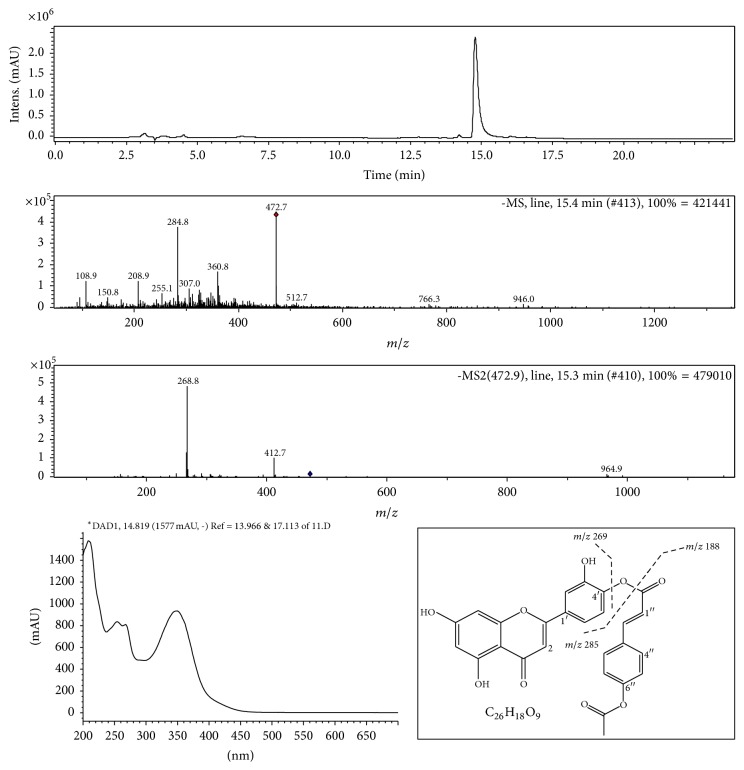
Chemical characterization of 4′-*p*-acetylcoumaroyl luteolin by RP-HPLC, UV, LC-ESI/MS, and ESI/MS2 using Agilent Zorbax 300 Extend C-18 Column (3.5 *µ*m, 4.6 mm ID × 150 mm).

The results of the* in vitro* and* in vivo* antioxidant activities showed promising effects of the ethyl acetate fraction and hence it was chosen for further purification. Fractionation by silica gel column chromatography and subsequent repurification of the most active subfractions by semipreparative HPLC led to the isolation of a potent antioxidant compound. The bioactive molecule structure was established based on the obtained spectroscopic data (^1^H- and ^13^C-NMR, UV, and MS) leading to the identification of a new compound: 4′-*p*-acetylcoumaroyl luteolin (Figure 4). The antioxidant activity of this compound, using DPPH test, showed a strong antioxidant activity with IC_50_ of 20.74 ± 0.6 *μ*g/ml. Furthermore, its AAI index of 1.92 was better than that of the BHT used as control. The amount of the 4′-*p*-acetylcoumaroyl luteolin in the ethyl acetate fraction was 0.89 (w/w).

## 4. Discussion

The oxidative stress caused by ROS overproduction, which cannot be overcome by human body, was considered as one of the causative agents of diseases. Serious health problems, such as cancer, cardiovascular disorder, inflammation, depression, and liver diseases, caused by proteins, lipids, and DNA oxidation have been reported worldwide [2]. Therefore, numerous attempts to evaluate the scientific basis of herbs and fruits rich in natural antioxidants have been made to reduce the serious effects of ROS [24]. The present study demonstrated that the different* R. tingitanus* leaves extracts exhibited a strong antioxidant capacity on oxidative stress and liver damage induced by CCl_4_ exposure in rats. Different* in vitro* physicochemical tests miming the efficacy of biological antioxidants to prevent cells dysfunction were used. The DPPH assay is a commonly used method in measuring the biomolecules' antioxidant activity [25]. The Rt EtOAcF and Rt EtOH-H_2_O exhibited a significantly higher DPPH scavenging activity than the other extracts. The antiradical potential of* R. tingitanus* leaves extracts is based on the potent-donating ability of* Rumex* phytoconstituents [26]. The reductive capacity of Rt EtOAcF and Rt EtOH-H_2_O which are higher than the reference antioxidant BHT confirmed the antioxidant properties of these extracts. Equally, the Rt EtOAcF and Rt EtOH-H_2_O are able to neutralize lipid hydroperoxides derived from linoleic acid and therefore protect unsaturated fatty acids and bleaching of *β*-carotene [27]. These results revealed the ability of Rt EtOAcF and Rt EtOH-H_2_O to produce more stable and nonreactive products from reactive free radicals and to stop their chain reactions by proton or electron donation. Thus, the richness of these two extracts in phenolic compounds, known for their chain-breaking and oxygen scavenging capacities, explains their antioxidant potential. Phenolic molecules are considered as one of the most beneficial compounds to prevent and treat many diseases especially those related to oxidative stress such as cancer, inflammatory cardiovascular, and neurodegenerative and liver diseases [28]. From the* in vitro* studies, there is good evidence that the Rt EtOAcF and Rt EtOH-H_2_O displayed potent antioxidant activities. Extrapolating the* in vitro* data to predict the protective effect of Rt EtOH-H_2_O against the CCl_4_-induced hepatotoxicity has become essential. The trichloromethyl radical (CCl_3_^*∗*^) and peroxy trichloromethyl radical (CCl_3_OO^*∗*^) generated by CCl_4_ metabolism induce serious liver lipid and protein damage. The lipid peroxidation of the liver could increase some biochemical parameters like MDA level and decrease SOD, CAT, and GPx activities [29]. The increased levels of MDA and impaired antioxidant enzymes activities, as shown in our study, suggest membrane damage due to lipid peroxidation which is the main cause of hepatotoxicity [30]. Pretreatment with Rt EtOH-H_2_O significantly reversed these changes and enhanced the hepatic CAT, SOD, and GPx activities. These enzymes are involved in the cellular antioxidant defense mechanism through scavenging toxic intermediates of reactive oxygen species via a cascade of reactions. H_2_O_2_ generated by the conversion of the superoxide radicals by SOD was rapidly transformed into water by CAT and GPx [31]. Therefore, Rt EtOH-H_2_O displayed a potent capacity to scavenge the reactive species leading to protection of liver from increased MDA formation and aided in maintaining the antioxidant enzyme activities. The investigation of the acute toxicity of* Rumex* extracts, after administration of different Rt EtOH-H_2_O concentrations, revealed that there is no behavioral or physiological change in treated mice after 7 days. Therefore,* Rumex* could be considered as safe and a promising candidate in phytomedicine.

Decreased SOD, CAT, and GPx activities and elevated MDA levels have been identified in patient with recurrent depression [32]. In this context, the current study intended to evaluate the antidepressant-like proprieties of the hydroalcoholic and ethyl acetate extracts obtained from* R. tingitanus *using the FST in mice. The results showed that the preventive treatment with these two extracts is effective to induce a significant antidepressant-like response. Noteworthy,* R. tingitanus *extracts caused a decrease in the immobility time similar to clomipramine effect used as a classical antidepressant. In this regard, medical therapies with* R. tingitanus *may be effective alternatives to conventional antidepressants which have a high economic cost and several side effects.

The observed* in vitro* effects that are confirmed also* in vivo*, achieved by Rt EtOH-H_2_O and Rt EtOAcF, correlated with their richness in phenolics and flavonoids known for their beneficial effects on human health [33]. Thus, Rt EtOAcF was subject to bio-guided fractionation and identification of the most potent antioxidant compound. The structure of the isolated antioxidant compound was established by ^1^H NMR, ^13^C NMR, UV, RP-HPLC, and LC-ESI-MS/MS. A new isolated compound was identified as 4′-*p*-acetylcoumaroyl luteolin (Figure 4). The luteolin was previously isolated from this plant [10] and reported in three other* Rumex* species, the root of* R. hastatus* [34] and* R. pictus* and* R. vesicarius* leaves [35]. Also, glycosylated luteolin, such as luteolin-7-O-glucoside and luteolin-8-C-glucoside, was described in other genus species [7]. The 4′-*p*-acetylcoumaroyl luteolin showed a strong antioxidant activity but it was lesser than luteolin. The difference between the two compounds could be attributed to the substitution of the OH in luteolin by a* p*-acetylcoumaroyl group. The* p*-coumaroyl moiety diminished the radical scavenging activity [36]. The edible plant* R. tingitanus* showed a bioprotective effect* in vitro* and* in vivo* against ROS and an antidepressant like-effect.

## 5. Conclusion

In summary, this work provided new findings and insights about the pharmacological activities, toxicity, and chemical constituents of* Rumex tingitanus*. From the* in vitro* studies, there is good evidence that the Rt EtOAcF and Rt EtOH-H_2_O displayed potent antioxidant activities. The* in vivo* study confirmed the* in vitro* results. The treatment with the* Rumex tingitanus* extracts showed a hepatoprotective effect against oxidative damage induced by CCl_4_ thanks to its phenolic composition. Moreover,* R. tingitanus* can prevent depression disorders as proven by the forced swimming test. A new compound* 4*′-*p*-acetylcoumaroyl luteolin with a potent antioxidant activity was isolated from the ethyl acetate fraction. Hence, our results suggest a basis for the possible use of* R. tingitanus *leaves as a potential new drug for acute liver injury and depression diseases. This study thus provides new insights for further promising investigations of* Rumex* species and their bioactive molecules to the development of pharmacological targets to reduce the marked adverse effects of chemical agents.

## Figures and Tables

**Table 1:** The IC_50_ and AAI values of DPPH test and *β*-carotene-linoleic acid assay and IC_0.5_ values of reducing power assay of *R. tingitanus *leaves extracts.

Extract	DPPH		*β*-Carotene	Reducing power
	IC_50_ (*µ*g/ml)	AAI	IC_50_ (*µ*g/ml)	IC_0.5_ (*µ*g/ml)
Rt EtOH-H_2_O	78.1 ± 1.5	0.51	451 ± 9	106 ± 3.1
Rt EtOAcF	11.7 ± 0.2	3.39	320 ± 16	41 ± 2
Rt HexF	193.8 ± 7.9	0.26	>2000	410 ± 24.6
Rt WF	81.4 ± 4	0.49	>2000	133 ± 7.9
Luteolin	12.7 ± 0.2	3.12	19 ± 1	nt
4′-*p*-acetylcoumaroyl luteolin	20.7 ± 0.6	1.92	nt	nt
BHT	86.5 ± 1.7	0.46	5.01 ± 0.15	41 ± 2.1
Ascorbic acid	3.7 ± 0.2	10.61	9.60 ± 0.01	nt

BHT and ascorbic acid were used as standard; IC_50_ (*µ*g/mL): values corresponding to the extract amount required to scavenge 50% of radicals present in the reaction mixture; IC_0.5_: extract or compound concentration providing absorbance of 0.5; AAI: antioxidant activity index; nt: not tested.

**Table 2:** Effects of CCl_4_, Rt EtOH-H_2_O, GA, and their combinations (Rt EtOH-H_2_O/CCl_4_ and GA/CCl_4_) on hepatic MDA and the enzymatic antioxidant activities in liver of control andexperimental rats.

Treatment	MDA (nmol/mg protein)	SOD (IU/mg protein)	CAT (IU/mg protein)	GPx (IU/mg protein)
Normal control	1.27 ± 0.02	23 ± 3.10	1049 ± 61	3.40 ± 1.03
CCl_4_ control	1.90 ± 0.03^c^	12.8 ± 1.20^c^	509.5 ± 16^c^	0.74 ± 0.02^c^
Rt EtOH-H_2_O/CCl_4_	1.16 ± 0.04^*∗∗∗*^	23.6 ± 5.90^*∗∗∗*^	1043 ± 49^*∗∗∗*^	3.30 ± 0.17^*∗∗∗*^
GA/CCl_4_	1.30 ± 0.03^*∗∗∗*^	21.4 ± 1.10^*∗∗∗*^	1049 ± 30^*∗∗∗*^	3.35 ± 0.21^*∗∗∗*^
Rt EtOH-H_2_O	1.30 ± 0.01^*∗∗∗*^	23 ± 1.90^*∗∗∗*^	1016 ± 59^*∗∗∗*^	3.25 ± 0.13^*∗∗∗*^
GA	1.25 ± 0.02^*∗∗∗*^	21.8 ± 4.50^*∗∗∗*^	1077 ± 24^*∗∗∗*^	3 ± 0.28^*∗∗∗*^

Values are mean ± SE. *n* = 6 in each group: GA (gallic acid), Rt EtOH-H_2_O, CCl_4_, Rt EtOH-H_2_O/CCl_4_, and GA/CCl_4_ treated groups. Superscript c denotes statistical significance at *P* < 0.001 when compared to control group. Superscript *∗∗∗* indicates statistical significance at *P* < 0.001 in comparison to CCl_4_ group.

## References

[1] Cassani J., Ferreyra-Cruz O. A., Dorantes-Barrón A. M., Vigueras Villaseñor R. M., Arrieta-Baez D., Estrada-Reyes R. (2015). Antidepressant-like and toxicological effects of a standardized aqueous extract of Chrysactinia mexicana A. Gray (Asteraceae) in mice. *Journal of Ethnopharmacology*.

[2] Sacchet C., Mocelin R., Sachett A. (2015). Antidepressant-like and antioxidant effects of plinia trunciflora in mice. *Evidence-Based Complementary and Alternative Medicine*.

[3] Michel T. M., Pülschen D., Thome J. (2012). The role of oxidative stress in depressive disorders. *Current Pharmaceutical Design*.

[4] Uttara B., Singh A. V., Zamboni P., Mahajan R. T. (2009). Oxidative stress and neurodegenerative diseases: a review of upstream and downstream antioxidant therapeutic options. *Current Neuropharmacology*.

[5] Jaeschke H., Gores G. J., Cederbaum A. I., Hinson J. A., Pessayre D., Lemasters J. J. (2002). Mechanisms of hepatotoxicity. *Toxicological Sciences*.

[6] Hardin B. L. (1954). Carbon tetrachloride poisoning. *Indian Journal of Surgery*.

[7] Vasas A., Orbán-Gyapai O., Hohmann J. (2015). The Genus Rumex: Review of traditional uses, phytochemistry and pharmacology. *Journal of Ethnopharmacology*.

[8] Zhang H., Guo Z., Wu N. (2012). Two novel naphthalene glucosides and an anthraquinone isolated from rumex dentatus and their antiproliferation activities in four cell lines. *Molecules*.

[9] Ismail H. B. (2013). Edible wild vegetables used in North West of Tunisia. *Paripex-Indian-Journal of Research*.

[10] Mhalla D., Bouaziz A., Ennouri K. (2017). Antimicrobial activity and bioguided fractionation of Rumex tingitanus extracts for meat preservation. *Meat Science*.

[11] De S., Dey Y. N., Ghosh A. K. (2010). Pytochemical investigation and chromatographic evaluation of the different extracts of tuber of Amorphaphallus paeoniifolius (Araceae). *International Journal on Pharmaceutical and Biomedical Research*.

[12] Mueller P. (1994). *Analysis of Phenolic Plant Metabolites*.

[13] Quettier-Deleu C., Gressier B., Vasseur J. (2000). Phenolic compounds and antioxidant activities of buckwheat (Fagopyrum esculentum Moench) hulls and flour. *Journal of Ethnopharmacology*.

[14] Adewale O. B., Onasanya A., Anadozie S. O. (2016). Evaluation of acute and subacute toxicity of aqueous extract of Crassocephalum rubens leaves in rats. *Journal of Ethnopharmacology*.

[15] Can A., Dao D. T., Arad M., Terrillion C. E., Piantadosi S. C., Gould T. D. (2012). The mouse forced swim test. *Journal of visualized experiments : JoVE*.

[16] Trigui M., Hsouna A. B., Tounsi S., Jaoua S. (2013). Chemical composition and evaluation of antioxidant and antimicrobial activities of Tunisian Thymelaea hirsuta with special reference to its mode of action. *Industrial Crops and Products*.

[17] Oyaizu M. (1986). Studies on products of browning reaction: antioxidative activity of products of browning reaction prepared from glucosamine. *The Japanese Journal of Nutrition and Dietetics*.

[18] Beyer W. F., Fridovich I. (1987). Assaying for superoxide dismutase activity: some large consequences of minor changes in conditions. *Analytical Biochemistry*.

[19] Bergmeyer H. U., H. Aebi (1974). *Catalase, in Method of Enzymatic Analysis*.

[20] Flohe L., Gunzler W. A. (1984). Assays of glutathione peroxidase. *Methods in Enzymology*.

[21] Gabe M., Masson C. O. (1968). Les colorations. *Topographiques*.

[22] Ahmadi F., Sadeghi S., Modarresi M., Abiri R., Mikaeli A. (2010). Chemical composition, in vitro anti-microbial, antifungal and antioxidant activities of the essential oil and methanolic extract of Hymenocrater longiflorus Benth. of Iran. *Food and Chemical Toxicology*.

[23] Keller J. N., Mattson M. P. (1998). Roles of lipid peroxidation in modulation of cellular signaling pathways, cell dysfunction, and death in the nervous system. *Reviews in the Neurosciences*.

[24] Baranisrinivasan P., Elumalai E. K., Sivakumar C., Viviyan Therasa S., David E. (2009). Hepatoprotective effect of Enicostemma littorale blume and Eclipta alba during ethanol induced oxidative stress in albino rats. *International Journal of Pharmacology*.

[25] Awika J. M., Rooney L. W., Wu X., Prior R. L., Cisneros-Zevallos L. (2003). Screening methods to measure antioxidant activity of sorghum (Sorghum bicolor) and sorghum products. *Journal of Agricultural and Food Chemistry*.

[26] Halliwell B. (1991). Reactive oxygen species in living systems: source, biochemistry, and role in human disease. *American Journal of Medicine*.

[27] Kulisic T., Radonic A., Katalinic V., Milos M. (2004). Use of different methods for testing antioxidative activity of oregano essential oil. *Food Chemistry*.

[28] Mishra S., Aeri V., Katare D. P. (2014). Hepatoprotective medication for liver injury. *Journal of Pharmacy and Pharmaceutical Sciences*.

[29] Yao X., Zhu L., Chen Y., Tian J., Wang Y. (2013). In vivo and in vitro antioxidant activity and *α*-glucosidase, *α*-amylase inhibitory effects of flavonoids from Cichorium glandulosum seeds. *Food Chemistry*.

[30] Goel A., Dani V., Dhawan D. K. (2005). Protective effects of zinc on lipid peroxidation, antioxidant enzymes and hepatic histoarchitecture in chlorpyrifos-induced toxicity. *Chemico-Biological Interactions*.

[31] Sharma P., Jha A. B., Dubey R. S., Pessarakli M. (2012). Reactive oxygen species, oxidative damage, and antioxidative defense mechanism in plants under stressful conditions. *Journal of Botany*.

[32] Soares J. C., Young A. (2016). *Bipolar Disorders: Basic Mechanisms and Therapeutic Implications*.

[33] Hsiao G., Shen M., Lin K. (2003). Antioxidative and hepatoprotective effects of Antrodia camphorata extract. *Journal of Agricultural and Food Chemistry*.

[34] Sahreen S., Khan M. R., Khan R. A. (2014). Comprehensive assessment of phenolics and antiradical potential of Rumex hastatus D. Don. roots. *BMC Complementary and Alternative Medicine*.

[35] El-Hawary S. A., Sokkar N. M., Ali Z. Y., Yehia M. M. (2011). A Profile of Bioactive Compounds of Rumex vesicarius L.. *Journal of Food Science*.

[36] Braca A., Fico G., Morelli I., de Simone F., Tomè F., De Tommasi N. (2003). Antioxidant and free radical scavenging activity of flavonol glycosides from different *Aconitum* species. *Journal of Ethnopharmacology*.

